# Nuclear Expression of Dynamin 2 Is Associated With Tumor Aggressiveness in Bladder Cancer Patients: A Bioinformatics and Experimental Approach

**DOI:** 10.1002/cnr2.2133

**Published:** 2024-11-28

**Authors:** Mahdieh Razmi, Leili Saeednejad Zanjani, Mandana Rahimi, Roya Sajed, Sadegh Safaei, Zahra Madjd, Roya Ghods

**Affiliations:** ^1^ Oncopathology Research Center Iran University of Medical Sciences (IUMS) Tehran Iran; ^2^ Department of Pathology and Genomic Medicine, Sidney Kimmel Cancer Center Thomas Jefferson University Philadelphia USA; ^3^ Hasheminejad Kidney Center, Pathology Department Iran University of Medical Sciences (IUMS) Tehran Iran; ^4^ Department of Molecular Medicine, Faculty of Advanced Technologies in Medicine Iran University of Medicine Sciences (IUMS) Tehran Iran

**Keywords:** bladder cancer (BC), bioinformatics, dynamin 2 (DNM2), immunohistochemistry (IHC), tissue microarray (TMA)

## Abstract

**Background:**

Dynamin 2 (DNM2) is aberrantly expressed in different malignancies and exerts a function in tumor progression.

**Aims:**

This study, for the first time, aimed to evaluate the clinical and prognostic value of DNM2 in the pathophysiology of bladder cancer using bioinformatics analysis and experimental evaluation.

**Methods and Results:**

We analyzed gene expression of DNM2 in bladder tumor by GEPIA2 and GENT2 platforms. Cluster subnetworks were recognized from the protein–protein interaction (PPI) network using the MCODE plugin to screen the key genes. Subsequently, the pathway enrichment analysis was evaluated. Then, the immunohistochemical examination was conducted on 209 paraffin‐embedded bladder cancer samples to determine the expression pattern and clinical importance of DNM2. Our data mining findings demonstrated dysregulation of DNM2 gene expression in bladder cancer. The results of pathway and PPI network analyses indicated that DNM2 might be involved in the development of bladder cancer by influencing various signaling pathways. Our IHC results represented remarkably higher DNM2 expression in bladder tumor samples compared to normal tissue samples adjacent to tumor. A statistically significant association was identified between DNM2 expression in the nucleus and higher histological grade (*p* = 0.026), advanced pT stage (*p* = 0.016), muscular invasion (*p* = 0.007), tumor recurrence (*p* = 0.030), and distant metastasis (*p* < 0.001). Moreover, the nuclear DNM2 expression was observed to have prognostic significance for disease‐specific survival (DSS) using a log‐rank test (*p* = 0.028).

**Conclusion:**

These findings suggest that nuclear DNM2 expression could be a putative indicator of bladder tumor progression owing to its association with elevated cancer aggressiveness.

## 1 Introduction

Bladder cancer (BC) is the most common malignancy of the urinary tract worldwide, with approximately 549 000 new cases a year [1, 2]. The most frequent histological type of BC is urothelial cell carcinoma, which is also called transitional cell carcinoma (TCC) [3]. Bladder tumor is clinically classified into two distinct subtypes; approximately 75% of cases possess nonmuscle‐invasive BC (NMIBC), which is frequently recurrent, and 10%–30% of cases develop a progression to muscle‐invasive BC (MIBC), which advances a distant metastasis after radical cystectomy and leads to a lower survival [4, 5]. Despite advances in treatment strategies, the long‐term prognosis of invasive bladder tumor cases remains poor, and the molecular mechanisms underlying BC development remain unclear [6]. Therefore, investigation of the molecular pathogenesis of bladder tumor progression and exploration of valuable diagnostic and prognostic biomarkers are urgently warranted, with the aim of providing novel targeted molecular therapies.

Several studies have recently demonstrated dynamin 2 (DNM2) as a promising prognostic biomarker and therapeutic target of different cancers because of its involvement in a variety of pro‐oncogenic processes [7]. DNM2 is a 100 kDa multi‐domain protein and belongs to the large microtubule‐associated GTPase dynamin superfamily. While two other dynamin isoforms exhibit tissue‐specific expression, DNM2 is ubiquitously expressed in various tissues [8, 9]. Historically, DNM2 is realized for its broad physiological functions in various processes, including vesicle formation and trafficking and interaction with microtubules [10, 11, 12]. However, recent reports have cleared that overexpression of DNM2 stimulates cell proliferation, migration, invasion, and metastasis of various kinds of carcinomas [7].

Oncogenic mechanisms underlying elevated expression of DNM2 are relatively revealed. DNM2 is involved in cell division, signaling, and matrix metalloproteinase secretion, all of which have been involved in the progression of a range of malignancies [13, 14]. In addition, DNM2 exerts a significant function in vascular endothelial growth factor (VEGF)‐dependent angiogenesis [15] and homology‐directed repair (HDR)‐related chemoresistance [11, 16]. DNM2 is also a potential cancer therapy target, as inhibiting it with GTPase inhibitors can suppress cellular processes like proliferation, invasion, and response to therapy in cervical [12] and pancreatic cancers [17]. Additionally, high DNM2 expression has been reported in different cancers [8], and its association with worse prognosis in cancers like thyroid [18] and prostate [17] and increased relapse risk in leukemia [19] and TNBC [11]. Our recent studies have also indicated the relationship between DNM2 overexpression and an aggressive phenotype of breast cancer [20] and clear cell renal cell carcinoma [21]. While the clinicopathological importance of DNM2 in the progression of some specific carcinomas has been well investigated, there is less research on the protein expression pattern in other tissues, like bladder cancer.

At the first stage of the current study, a bioinformatics analysis utilizing oncology databases was performed to investigate the importance of DNM2 in bladder cancer patients, as well as to understand the underlying mechanisms that contribute to tumor invasiveness. However, as research on the protein expression pattern of DNM2 in bladder cancer is less, we designed the current study, for the first time, to explore the membranous, cytoplasmic, and nuclear expression patterns of DNM2 in a series of transitional cell carcinoma tissues, as the most frequent histological type of bladder tumor, through immunohistochemistry (IHC) method on tissue microarray (TMA) slides. Then, the association of DNM2 expression with the clinical features and possible prognostic outcomes was assessed.

## 2 Materials and Methods

### 2.1 Data Mining

Gene Expression Profiling Interactive Analysis (GEPIA2) and Gene Expression database of Normal and Tumor tissues 2 (GENT2) platforms were applied to evaluate the altered gene expression of DNM2 in cancer and normal tissues. As valuable resources for gene expression analyses, whereas GEPIA2 (http://gepia2.cancer‐pku.cn/#degenes) is collected from the “The Cancer Genome Atlas” (TCGA) and the “Genotype‐Tissue Expression” (GTEx) resources [22], The NCBI‐GEO database is used for GENT2 (http://gent2.appex.kr) with the Affymetrix U133A and U133Plus2 microarrays [23]. Moreover, the UALCAN platform was applied to assess protein expression data for DNM2 expression from bladder cancer patients. UALCAN (http://ualcan.path.uab.edu/), as a user‐friendly database, enables assessing tumor OMICS data from TCGA and Clinical Proteomic Tumor Analysis Consortium (CPTAC) [24]. In the following step, the STRING web‐based tool (confidence score: 0.9) was applied to identify potential DNM2 protein–protein interactions (PPIs) network virtualized by Cytoscape software version 3.9.1 [25]. A clustering analysis was conducted to extract hub genes in different signaling pathways through the Cytoscape plugin Molecular Complex Detection (MCODE) (degree cut‐off: 2, node score cut‐off: 0.2, and K‐Core: 2) [26], and the proteins in the cluster involving DNM2 were extracted for enrichment analysis. Finally, the resulting molecules were subjected to Enrichr (amp.pharm.mssm.edu/Enrichr/) and ClueGO/CluePedia to evaluate the significantly top‐related pathways involving DNM2 in Reactome [27] and BioPlanet [28]. *p* values <0.05 were considered to be significant. Additionally, to evaluate the subcellular localization of DNM2, the GeneCards database (https://www.genecards.org) was used [29]. Finally, we investigated the protein expression of DNM2 in different stages through the immunohistochemical method in bladder cancer tissue cases.

### 2.2 Patient Characteristics and Tumor Samples

In the current research, a total of 225 formalin‐fixed, paraffin‐embedded (FFPE) tissues of bladder cancer cases were enrolled between 2008 and 2011 from Hasheminejad Hospital in Tehran, Iran. All cases were enrolled from patients who had experienced transurethral resection of bladder tumor (TURB), and radiotherapy or chemotherapy was not used for them before the surgery. The archived patients' documents were retrieved to collect patients' clinical and pathological parameters, including age, gender, grade, tumor size (maximum tumor diameter), pT stage, muscularis propria invasion, distant metastasis, and tumor recurrence. Further, eight normal tissue samples adjacent to tumor were utilized to assess the DNM2 expression pattern compared to cancerous tissues. The pT stage was determined according to the pTNM staging system [30, 31]. We employed a classification system based on the International Society of Urological Pathology (ISUP) to categorize cases into high grade and low grade classifications [32]. In addition, information regarding patient outcomes, including overall survival (OS), which represents the period from the surgical procedure until either the date of death or the last follow‐up visit, disease‐specific survival (DSS), which determines as the interval from resection to cancer‐caused death, and progression‐free survival (PFS), which defines as the time of the surgery until the progression of the disease, were collected. Informed consent was received from participants who contributed samples to the study. This study has been approved by the Human Research Ethics Committee of Iran University of Medical Sciences in Iran (Ref No: 98‐04‐28‐17448).

Furthermore, data concerning the well‐being of patients, such as their OS, which measures the period from the surgical procedure to either the date of death or the most recent check‐up, DSS, which calculates the time from the tumor removal to death caused by cancer, and PFS, which indicates the duration from surgery until the disease progresses, were collected. This information was obtained after providing patients with necessary details and obtaining their consent.

### 2.3 Tissue microarray Construction

A precision arraying instrument (Tissue Arrayer Minicore; ALPHELYS, Plaisir, France) was used to prepare BC TMAs, as described previously [33, 34]. Briefly, pathologists assessed H&E slides to point to three representative tissue regions of the tumor in each block. Then, after punching out (0.6 mm diameter), exact annotated parts of each block were placed in the recipient TMA blocks. In the current research, at least three cores were assessed for each sample and then scored individually to control the issue of heterogeneous expression of tumor antigens and augment the accuracy and validity of analyses [35, 36]. For each tissue, the overall score was calculated from the mean of the three cores.

### 2.4 Immunohistochemistry Staining

The DNM2 protein expression was assessed through our IHC laboratory procedure [37, 38, 39]. In brief, the TMA slides were dewaxed and then rehydrated under ethanol treatment. Then, endogenous peroxidase activity blocking was performed at room temperature using 3% hydrogen peroxide solution for 20 min. Subsequently, after washing the sections with Tris Buffered Saline (TBS), heat‐activated antigen retrieval was done by autoclaving the slides in Tris‐EDTA buffer (pH 9) for 10 min. Following washing in TBS, sheep serum (5%) diluted in blocker protein (Dako, Denmark) was applied to block nonspecific binding sites for 20 min. Then, the primary antibody against dynamin 2 (ab3457, Abcam, USA, 100 ng/mL) was added to each section at 4°C overnight. The next day, the Mouse/Rabbit PolyVue HRP/DAB Detection kit (standard EnVision‐HRP kit, Bio pharmadx) as the secondary antibody was utilized for incubation of TMA sections for 1 h. Afterwards, sections were stained with 3,3′‐diaminobenzidine (DAB) chromogen substrate and counterstained with hematoxylin (Dako, Denmark). Ultimately, the dehydration of the slides was performed using graded alcohol, cleared in xylenes (Dako), and mounted. Normal whole tissue samples adjacent to tumor were utilized as a positive control. Primary antibody was replaced by rabbit immunoglobulin (rabbit IgG, 100 ng/mL) in negative reagent control [20, 37, 40].

### 2.5 Evaluation of Immunostaining

DNM2 immunostaining was assessed through a semi‐quantitative scoring system by two pathologists who were blinded to all clinicopathological data. A semi‐quantitative system was applied to score the intensity of staining in a range from negative to strong (0 [negative], 1 [weak], 2 [moderate], and 3 [strong]). The percentage of positively stained neoplastic cells was scored from 0% to 100% and, subsequently, classified based on the positively stained cells (1 [<25%], 2 [25%–50%], 3 [51%–75%], and 4 [>75%]). Then, the histochemical score (H‐score) was acquired by multiplying the score of intensity (0–3) and the percentage of stained tumor cells (0%–100%), yielding a score of 0 to 300 for each core. The final score was calculated from the mean of the three cores. In the present research, cut‐off points (150 and 285) were opted on the basis of the median H‐scores to classify the specimens as high or low membranous and cytoplasmic DNM2 expressions, respectively. For nuclear DNM2 expression, patients were classified into either a positive expression group (H‐score 1–300) or a negative expression group (H‐score 0).

### 2.6 Statistical Analysis

Statistical software SPSS, version 22.0 (IBM Corp, USA), was applied to perform quantitative analysis. In the present research, the classified data were represented by *N* (%) and quantitative data by mean (SD) and median (Q1, Q3). Furthermore, the significance of correlation and association between the DNM2 expression and clinical parameters was assessed using Spearman's correlation and Pearson's *χ* ^2^ analyses. The Kaplan–Meier plots were drawn for DSS and PFS, and then log‐rank analysis was applied to compare survival between groups. To clarify which variables influenced survival outcomes, the Cox proportional hazards regression analysis was utilized. Multivariable analyses were performed for variables influencing survival in univariate analysis. *p* values less than 0.05 were defined as statistically significant.

## 3 Results

### 3.1 Bioinformatics Approaches

**FIGURE 1:**
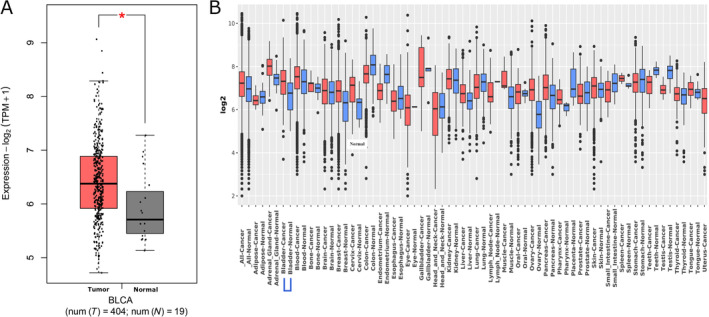
The mRNA dynamin 2 (DNM2) expression in bladder cancer by Gene Expression Profiling Interactive Analysis 2 (GEPIA2) and the Genotype‐Tissue Expression (GTEx) resources. The box plot expression results by GEPIA2 indicated overexpression of the DNM2 gene in tumor samples compared to normal samples (|Log2FC| Cut‐off: 0.5 and *p* < 0.01) (A). GENT2 database showed that the DNM2 gene expression level was significantly higher in the bladder tumor (GPL570 platform) sample in comparison with the normal samples (|Log2FC|: 0.607 and *p* < 0.001) (B).

GEPIA2 analysis by the TCGA database revealed a significant increase in DNM2 mRNA expression (|Log2FC| Cut‐off: 0.5) in 404 bladder tumor tissues in comparison with 19 normal bladder samples (*p* < 0.01, Figure 1A). Further, we identified significantly higher GEO gene expression of DNM2 in bladder cancer cases compared to normal bladder cases according to GENT2 (GPL570 platform) (*p* < 0.001, Figure 1B and Table S1).

**FIGURE 2:**
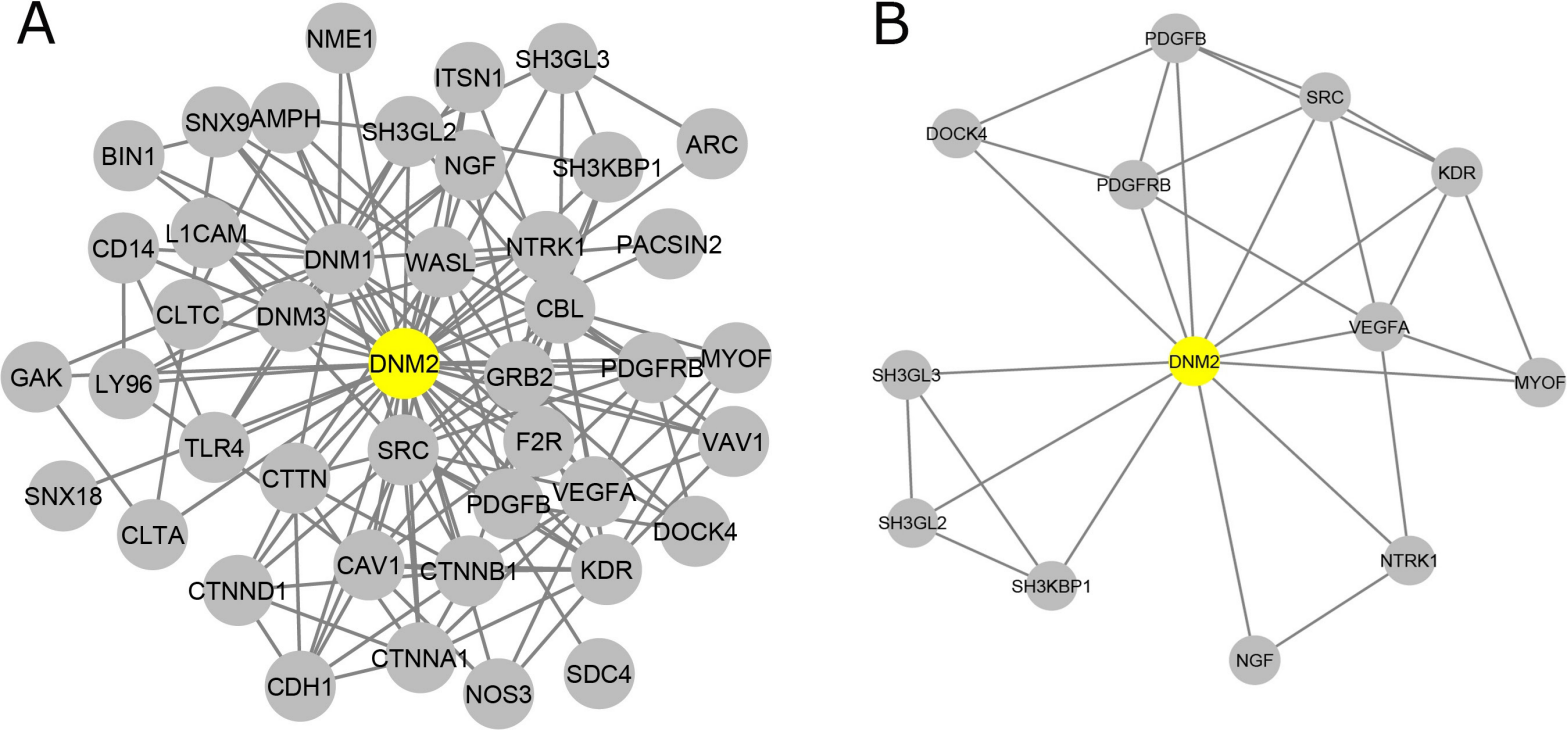
Protein–protein interaction (PPI) network and clusters for dynamin 2 (DNM2) obtained from the STRING tool and virtualized through Cytoscape software. (A) PPI network analysis by the STRING database revealed the interactions of DNM2 with other genes with the highest degree of connectivity (confidence score >0.9). The network includes 43 nodes and 159 edges of PPI. (B) The first cluster subnetwork was recognized from the PPI network using Cytoscape with the help of the MCODE plugin. The cluster includes 13 nodes and 29 edges.

**FIGURE 3:**
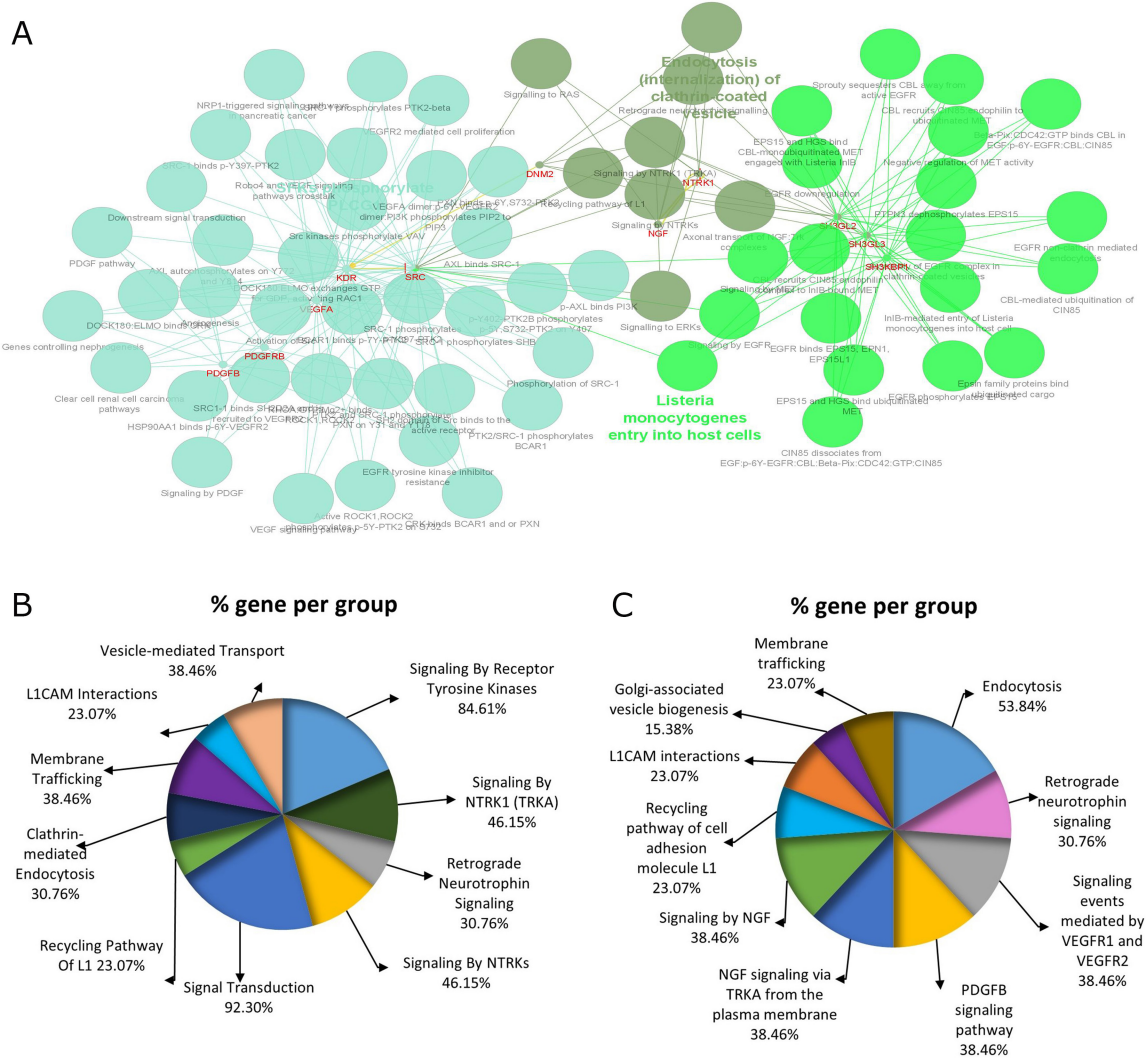
Pathway enrichment analysis for 13 extracted genes by the ClueGO/CluePedia plugin from Cytoscape with *p <* 0.05 (A) and the Enrichr tool using (B) Reactome and (C) BioPlanet databases with *p <* 0.0001.

Figure 2A shows the network with 43 nodes and 159 edges of PPI. Two clusters were obtained via the Cytoscape plugin MCODE and represented graphically in Figures 2B and S1. A Cluster with 13 nodes and 29 edges involving DNM2 was selected for subsequent analysis (Figure 2B). To a better understanding of the characteristics of these genes, the Cytoscape plugin ClueGO/CluePedia and Enrichr tool were applied to carry out pathway enrichment analysis of the identified PPI complex network (Figure 3A). Taken together, the top 10 results of pathway analysis by Reactome and BioPlanet in the Enrichr tool, in which DNM2 was involved, were shown in Figure 3B,C (*p* < 0.0001).

Subcellular localization assessment of DNM2 represented that it is mostly localized in the membrane, cytosol, and extracellular space, and also, to some extent, it is predicted to be found in the cell nucleus based on GeneCards database (Figure S2). Further, ULCAN database demonstrated that protein expression of DNM2 was not assessed in bladder cancer tissues.

### 3.2 Characteristics of Bladder Cancer Patients

The present study included 225 BC cases, and, finally, 209 bladder cancer tissues remained for assessment due to a loss of some cases. The calculated median age of cases was 66 years (SD = 13.27, range 25–95). For clarification, 106 (50.7%) were younger than 66 years old, and 103 (49.3%) were over 66 years old. The TMA specimens included 156 (74.6%) male and 53 (25.4%) female cases, with a male/female ratio of 2.94. A mean tumor size of 2.40 was obtained, ranging from 1 to 13 cm. One hundred and twenty‐eight (61.2%) of cases contained tumors less than the mean size (size ≤2.40), and 81 (38.8%) included cancers higher than the mean (size >2.40). In the current research, 126 (60.3%) cancerous tissues had a low grade, and 83 (39.7%) tumors contained a high grade. Additionally, 120 (57.4%) patients had stage 0, 71 (34.0%) stage I, and 18 (8.6%) stage II. Moreover, muscular invasion was found in 18 (8.6%) cases, respectively. Tumor recurrence was observed in 41 (19.6%) cases, and 25 (12.0%) patients exhibited distant metastasis during the follow‐up period. The patients' clinicopathological features are presented in Table 1.

**TABLE 1:** Patients and pathological characteristics of bladder carcinoma tissues.

Patients and tumor characteristics	Total samples *N*
Number of patients	209
Median age, years (range)	66 (25–95)
66≤	106
66>	103
Gender	
Male	156
Female	53
Tumor size (cm)	
Size ≤2.40	128
Size >2.40	81
Histological grade	
Low	126
High	83
pT stage	
pTa	120
pT1	71
pT2	18
pT3	0
pT4	0
Muscularis invasion	
Involved	18
None	191
Tumor recurrence	
Present	41
Absent	168
Distant metastasis	
Present	25
Absent	184

*Note: N* exhibits number of cases.

### 3.3 Expression of Dynamin 2 in the Bladder Cancer Tissues

**FIGURE 4:**
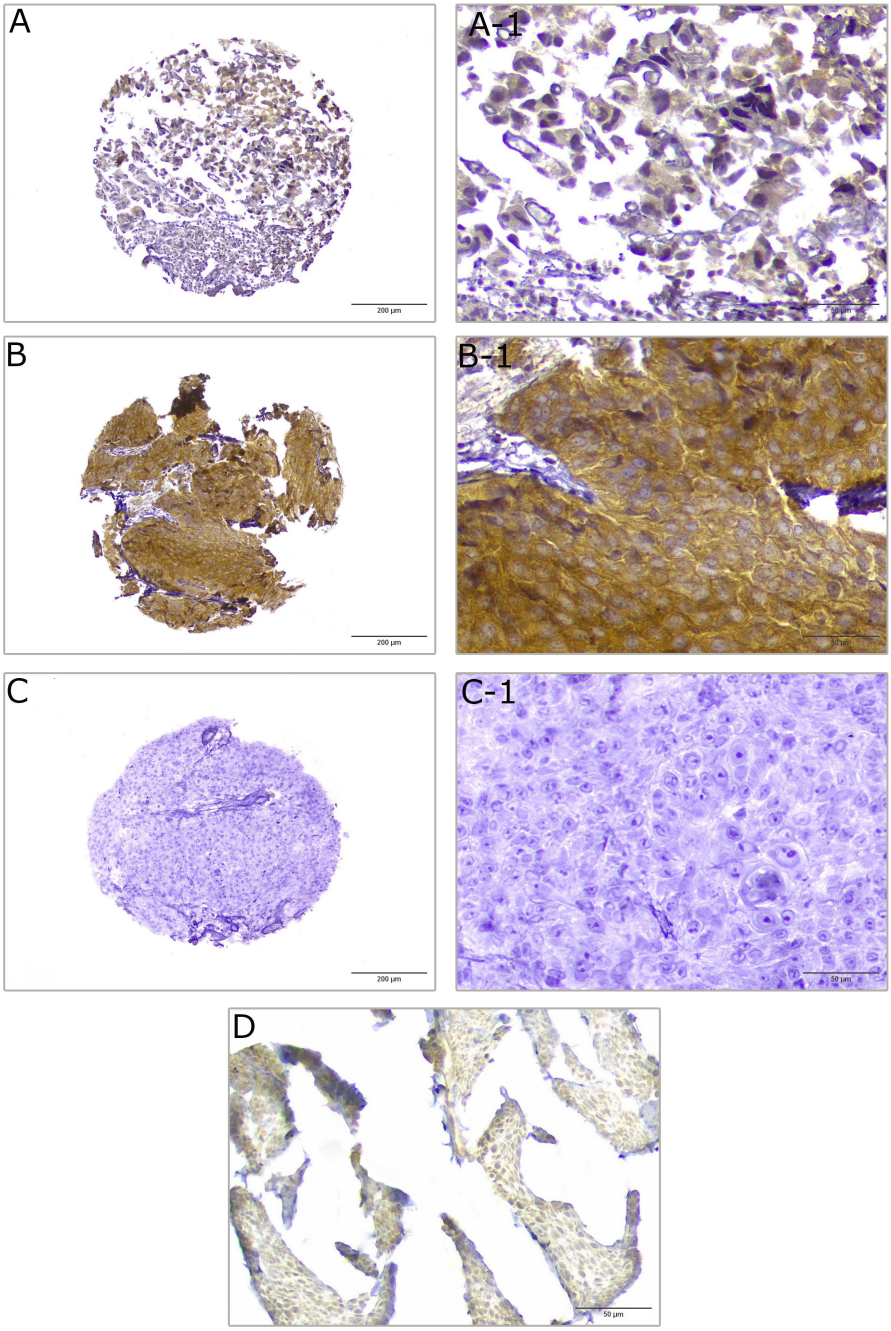
Immunohistochemical test of dynamin 2 (DNM2) expression in bladder tumor and normal tissue sample adjacent to tumor. Low expression (A, A‐1), high expression (B, B‐1), negative control (C, C‐1), and normal tissue adjacent to tumor (D). (A, B, and C): 100×, (A‐1, B‐1, C‐1, and D): 400×.

The protein expression level of DNM2 was determined through IHC on TMA sections on the basis of intensity, the percentage of positively stained cancer cells, and the H‐score. In the bladder tumor tissues, DNM2 was expressed at varying intensities in the cell membrane, cytoplasm, and nucleus (Figure 4). In terms of intensity, membranous, cytoplasmic, and nucleus expressions of DNM2 were recognized in 182 (87.1%), 207 (99.0%), and 27 (12.9%) bladder cancer cases, respectively (Table 2). Cytoplasmic DNM2 expression on the basis of the median H‐score (=285) as the cut‐off demonstrated that while 119 (56.9%) cases expressed low levels, 90 (43.1%) bladder cancer samples showed high levels of cytoplasmic expression of DNM2. Low and high membranous expressions of DNM2 (cut‐off = 150) were found in 108 (51.7%) and 101 (48.3%) patients, respectively (Table 2). For nuclear DNM2 expression, patients were categorized into either a positive expression group (27/209, 12.9%) or a negative expression group (182/209, 87.1%). Table 2 demonstrates the expression levels of this molecule. In addition, the results of IHC analysis for DNM2 exhibited membranous and cytoplasmic expression patterns in normal tissue samples adjacent to tumor. However, overexpression of DNM2 was found more in cancer tissues than in adjacent normal tissue samples with weak staining (Figure 4). The negative control figure is also presented in Figure 4.

**TABLE 2:** Expression of dynamin 2 (DNM2) protein (intensity of staining, percentage of positive tumor cells, and H‐score) in bladder carcinoma tissue samples.

Scoring system	Nuclear expression *N* (%)	Cytoplasmic expression *N* (%)	Membranous expression *N* (%)
Intensity of staining			
Negative (0)	182 (87.1)	2 (1.0)	27 (12.9)
Weak (+1)	9 (4.3)	25 (12.0)	17 (8.1)
Moderate (+2)	5 (2.4)	43 (20.5)	47 (22.5)
Strong (+3)	13 (6.2)	139 (66.5)	118 (56.5)
Percentage of positive tumor cells			
0%–25%	201 (96.2)	4 (1.9)	59 (28.2)
25%–50%	7 (3.3)	4 (1.9)	26 (12.4)
51%–75%	1 (0.5)	10 (4.8)	62 (29.7)
>75%	0 (0.0)	191 (91.4)	62 (29.7)
H‐score (cut off)			
	Positive or negative	Median (285)	Median (150)
	Negative 182 (87.1)	Low 119 (56.9)	Low 108 (51.7)
	Positive 27 (12.9)	High 90 (43.1)	High 101 (48.3)
Total	209	209	209

*Note:* H‐score indicates histological score.

### 3.4 Associations Between Expression of Dynamin 2 Protein and Clinicopathological Parameters in Bladder Cancer Patients

The Pearson's *χ* ^2^ analysis results represented a statistically significant association between nuclear expression of DNM2 and the increased grade (H‐score *p* = 0.026), advanced pT stage (H‐score *p* = 0.016), muscular invasion (H‐score *p* = 0.007), tumor recurrence (intensity *p* = 0.030), and distant metastasis (intensity *p* < 0.001) (Table 3). In addition, nuclear expression of DNM2 was directly correlated with grade (*p* = 0.026), advanced tumor stage (*p* = 0.021), and muscular invasion (*p* = 0.007) based on Spearman's correlation test. However, Spearman's analysis results did not show a considerable correlation between nuclear DNM2 expression and tumor recurrence (*p* = 0.202), and distant metastasis (*p* = 0.398).

**TABLE 3:** The association between nuclear dynamin 2 (DNM2) expression and clinicopathological characteristics in bladder carcinoma (intensity of staining and H‐score).

Patients and tumor characteristics	Total samples *N* (%)	Intensity of staining *N* (%)				*p*	H‐score (cut off = positive or negative) *N* (%)		*p*
		0 (negative)	1+ (weak)	2+ (moderate)	3+ (strong)		Negative	Positive	
Median age (years)									
66≤	106 (50.7)	96 (52.7)	2 (22.2)	1 (20)	7 (53.8)	0.160	96 (52.7)	10 (37.0)	0.128
66>	103 (49.3)	86 (47.3)	7 (77.8)	4 (80)	6 (46.2)		86 (47.3)	17 (63.0)	
Gender									
Male	156 (74.6)	134 (73.6)	7 (77.8)	4 (80)	11 (84.6)	0.824	134 (73.6)	22 (81.5)	0.381
Female	53 (25.4)	48 (26.4)	2 (22.2)	1 (20)	2 (15.4)		48 (26.4)	5 (18.5)	
Tumor size (cm)									
2.4≤	128 (61.2)	114 (62.6)	5 (55.6)	2 (40)	7 (53.8)	0.677	114 (62.6)	14 (51.9)	0.283
2.4>	81 (38.8)	68 (37.4)	4 (44.4)	3 (60)	6 (46.2)		68 (32.5)	13 (48.1)	
Histological grade									
Low	126 (60.3)	115 (63.2)	2 (22.2)	2 (40)	7 (53.8)	0.067	115 (63.2)	11 (40.7)	**0.026**
High	83 (39.7)	67 (36.8)	7 (77.8)	3 (60)	6 (46.2)		67 (36.8)	16 (59.3)	
pT stage									
pTa	120 (57.4)	109 (59.9)	2 (22.2)	2 (40)	7 (53.8)	**0.04**	109 (59.9)	11 (40.7)	**0.016**
pT1	71 (34.0)	61 (33.5)	5 (55.6)	1 (20)	4 (30.8)		61 (33.5)	10 (37.0)	
pT2	18 (8.6)	12 (6.6)	2 (22.2)	2 (11.1)	2 (15.4)		12 (6.6)	6 (22.2)	
pT3	0 (0.0)	0 (0.0)	0 (0.0)	0 (0.0)	0 (0.0)		0 (0.0)	0 (0.0)	
pT4	0 (0.0)	0 (0.0)	0 (0.0)	0 (0.0)	0 (0.0)		0 (0.0)	0 (0.0)	
Muscularis invasion									
Involved	18 (8.6)	12 (6.6)	2 (22.2)	2 (40.0)	2 (15.4)	**0.018**	12 (6.6)	6 (22.2)	**0.007**
None	191 (91.4)	170 (93.4)	7 (77.8)	3 (60.0)	11 (84.6)		170 (93.4)	21 (77.8)	
Tumor recurrence									
Present	41 (19.6)	33 (18.1)	5 (55.6)	0 (0.0)	3 (23.1)	**0.030**	33 (18.1)	8 (29.6)	0.160
Absent	168 (80.4)	149 (81.9)	4 (44.4)	5 (100)	10 (76.9)		149 (81.9)	19 (70.4)	
Distant metastasis									
Present	25 (12)	20 (11)	5 (55.6)	0 (0.0)	0 (0.0)	**<0.001**	20 (11)	5 (18.5)	0.261
Absent	184 (88)	162 (89)	4 (44.4)	5 (100)	13 (100)		162 (89.0)	22 (81.5)	

*Note:* H‐score indicates histological score. *p* value; Pearson's *χ* ^2^ test. Values in bold are statistically significant.

Additionally, Spearman's correlation and Pearson's *χ* ^2^ analyses displayed no correlation or association between cytoplasmic or membranous expression of DNM2 and the clinical features in bladder cancer cases (Tables S2 and S3).

### 3.5 Prognostic Significance of Dynamin 2 Protein Expression in Bladder Cancer Tissues

From the 209 bladder cancer samples included in this study, 45 (21.5%) patients were positive for metastasis, recurrence, or cancer‐related death, whereas 164 (78.5%) cases had no history of these parameters. In 25 (12.0%) and 41 (19.6%) patients, distant metastasis and tumor recurrence appeared, respectively. Cancer‐related death was observed in 29 (13.8%), and the other cause of death occurred in 14 patients (6.6%) during the follow‐up period. The median and mean follow‐up durations were 83 (Q1, Q3; 78, 93) and 79 (SD = 19.6) months, ranging from 1 to 98 months.

### 3.6 Survival Outcomes According to the Dynamin 2 Expression in Bladder Cancer Tissues

**FIGURE 5:**
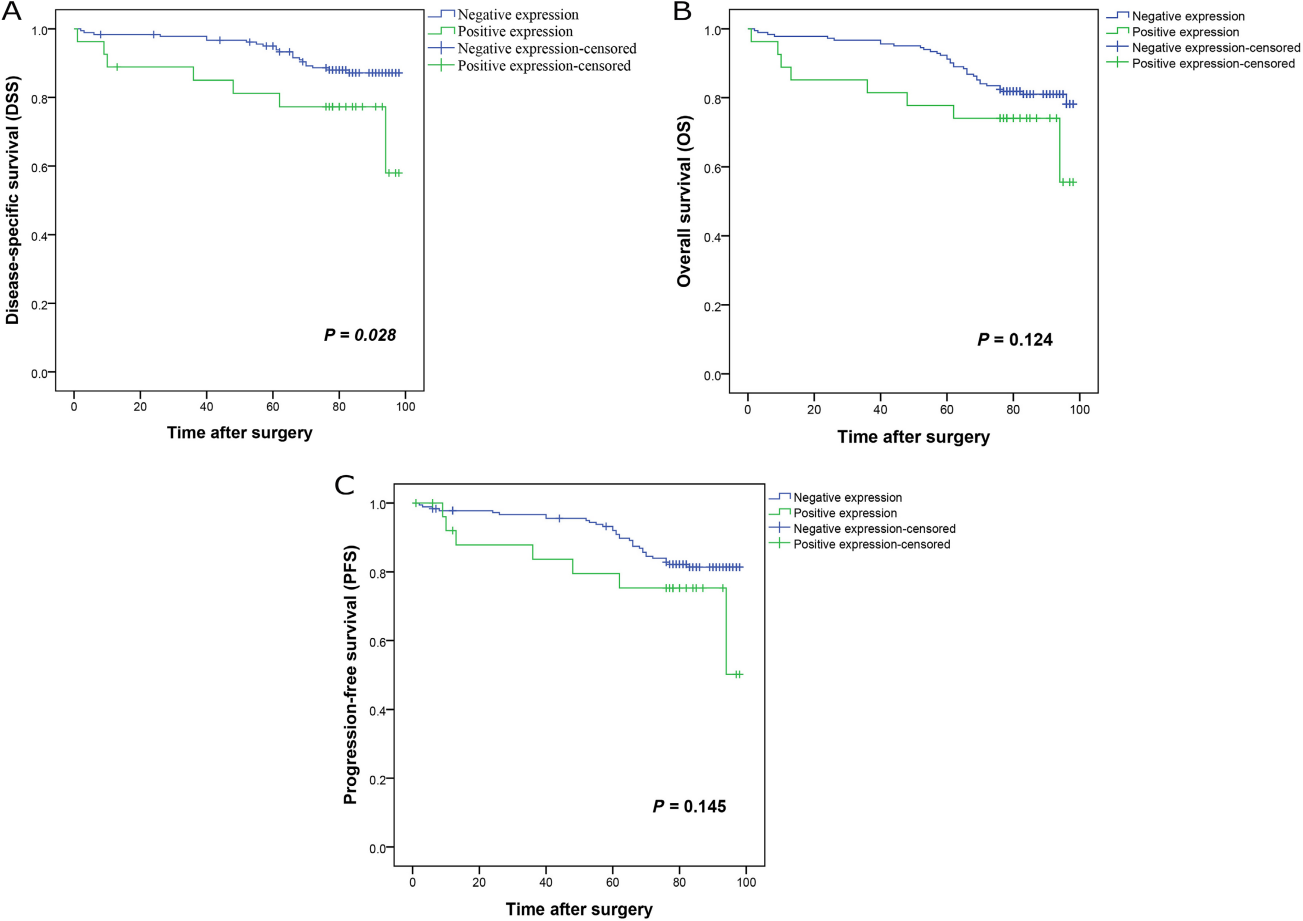
Kaplan–Meier curves for disease‐specific survival (DSS) (A), overall survival (OS) (B), and progression‐free survival (PFS) (C) according to the nuclear dynamin 2 (DNM2) protein expression level in bladder cancer patients. (A) Nuclear expression of DNM2 protein was associated with shorter DSS in bladder tumor patients than in cancers without expression of this molecule (*p* = 0.028). (B and C) Kaplan–Meier survival test demonstrated that nuclear DNM2 protein expression is not linked with OS (*p* = 0.124) and PFS (*p* = 0.145).

The Kaplan–Meier curve results displayed that bladder cancer cases with nuclear DNM2 expression had shorter DSS in comparison to cases without nuclear expression of DNM2 (Log Rank test, *p* = 0.028) (Figure 5A). The mean DSS times were 81 (SD = 5.9) and 92 (SD = 1.2) months for cases with positive and negative expression of nuclear DNM2, respectively. Additionally, the DSS 5‐year survival rates in cases with positive and negative nuclear expression of DNM2 were 77% and 89%, respectively (*p* = 0.05). However, there were no remarkable differences in OS and PFS of cases with positive and negative nuclear expression of DNM2 (*p* = 0.124 and *p* = 0.145, respectively) (Figure 5B,C).The mean times for PFS were 80 (SD = 6.2) and 89 (SD = 1.4) months for cases with positive and negative DNM2 nuclear expression, respectively.

Univariate and multivariate tests were utilized to investigate the importance of nuclear DNM2 expression and other clinicopathological features on survival outcomes in bladder cancer cases. As presented in Table 4, the results of univariate Cox regression analysis displayed that nuclear expression of DNM2 (*p* = 0.034), pT stage (*p* < 0.001), tumor differentiation (*p* = 0.002), and muscularis invasion (*p* < 0.001) were significant risk factors influencing DSS. Additionally, based on multivariate analysis, only muscularis invasion (*p* = 0.022) had a relationship with DSS, considering an independent prognostic factor for DSS (Table 4).

**TABLE 4:** Univariate and multivariate Cox regression analyses of potential prognostic factors for disease‐specific survival (in patients with bladder carcinoma).

Covariate	Univariate analysis		Multivariate analysis	
	HR (95% CI)	*p*	HR (95% CI)	*p*
Nuclear dynamin 2 expression (Positive versus negative)	2.506 (1.070–5.873)	***0.034***	1.726 (0.726–4.195)	0.213
Median age (years)	1.356 (0.652–2.820)	0.415	—	—
Gender	0.748 (0.305–1.838)	0.527	—	—
Tumor size (cm)	1.722 (0.831–3.569)	0.144	—	—
Histological grade (High grade versus low grade)	3.272 (1.519–7.049)	***0.002***	1.660 (0.457–6.031)	0.441
pT stage		***<0.001***	—	***0.04***
pT1 versus pTa	2.393 (1.007–5.682)	***0.048***	1.540 (0.404–5.875)	0.527
pT2 versus pTa	7.795 (3.000–20.259)	***<0.001***	4.477 (1.024–19.561)	***0.046***
Muscularis invasion (Involved versus none)	5.194 (2.295–11.752)	***<0.001***	2.907 (1.170–7.218)	***0.022***
Recurrence (Present versus absent)	0.554 (0.253–1.152)	0.114	—	—
Distant metastasis (Present versus absent)	0.621 (0.312–1.247)	0.245	—	—

*Note:* The variables with *p* value less than 0.05 in univariate analysis were included in multivariable analyses. Values in bold and italic are statistically significant.

Abbreviations: HR, hazard ratio; CI, confidence interval.

However, the Kaplan–Meier curves and univariate and multivariate analyses identified no considerable link between the cases with cytoplasmic and membranous DNM2 expression and DSS, OS, or PFS.

## 4 Discussion

Bladder tumor remains a main clinical challenge due to its worse prognosis, high incidence rate, and limited treatment options to minimize recurrence [41]. The oncogenesis of bladder cancer includes alterations in various oncogenes and multiple suppressor genes. Consequently, numerous molecular biomarkers can be applied to provide viable strategies to ameliorate tumor prognosis, risk stratification, and treatment [42].

DNM2, a microtubule‐associated large GTPase, was historically recognized as a protein involved in the regulation of vesicle transport and endocytosis trafficking [43, 44]. Aside from its physiological functions, recently published in vitro studies have shown that DNM2 is implicated in numerous tumorigenesis and tumor progression processes, including cancer cell proliferation, chemoresistance, cell migration, motility, invasion, and metastasis [13, 15, 45, 46, 47, 48, 49]. It has been reported that this protein forms direct interactions with Src, PI3K, and FAK to organize complexes that increase motility [50]. Additionally, high expression of DNM2 has been reported in a series of cancer types, including cervical [12], pancreatic [49], and prostate cancers [17]. Previous studies have exhibited that DNM2 has a remarkable association with cancer invasiveness [17], poor prognosis [19], and chemoresistance [11] in different cancers. Our previous findings have also demonstrated that nuclear DNM2 overexpression has a considerable relationship with pT stage, grade, and aggressiveness in breast cancer [20] and clear cell renal cell carcinoma [21]. Thus, the oncogenic function of DNM2 in a wide variety of biological investigations has proposed the importance of the DNM2 protein as a promising molecule in the targeted therapy of different cancers [8].

In our review of the literature, the clinicopathological importance of DNM2 protein in the urinary system, including bladder cancers, has obtained much less consideration compared to its expression and function in other tumors. Hence, this investigation is the first research to assess the protein expression pattern and clinicopathological value of DNM2 in a collection of BC tissue specimens conducted using IHC on TMA sections. To the best of our knowledge, only one study has investigated the gene expression levels of DNM2 on a series of 66 bladder cancer tissues through the RT‐PCR method. They suggested DNM2 as a potential risk factor for bladder cancer, however, without any information regarding expression pattern and prognosis [51].

In this study, data mining, using the omics data, identified significant overexpression of the DNM2 gene in bladder cancer. Furthermore, our study using the IHC method identified that DNM2 is overexpressed in bladder tumor tissues compared to normal tissue samples adjacent to tumor. Our investigation is in accord with a publication by Xu et al., which displayed a remarkable augmentation in DNM2 expression in the neoplastic epithelium of progressive prostate cancer (PCA) compared to adjacent normal prostate tissue [17]. We also found that DNM2 is principally localized in the cytoplasm and membrane, and nuclear localization is observed in only 13% of the bladder cancer tissues, as confirmed by in silico analysis. In agreement with our study, Ge et al. reported the weak nuclear localization of DNM2 in acute lymphoblastic leukemia [19]. In this regard, we had a significant number of early‐stage tumors with no metastasis in our total cohort, which may be an explanation for the absence of nuclear DNM2 expression in our overall population.

Importantly, our findings indicated that nuclear expression of DNM2 in the tissues is associated with prominent clinicopathological features, including advanced pT stage, grade, recurrence, and more metastatic and invasive carcinomas. In contrast, in cytoplasmic and membranous expressions, there was no association between DNM2 overexpression and clinicopathological parameters in BC patients. These results highlight the critical function of nuclear DNM2 deregulation in promoting bladder tumor progression. In agreement with our observations, Xu et al. represented that gene expression of DNM2 is significantly associated with higher grades and stages of bladder cancer [51]. Generally, the main aim of this study was to investigate the expression pattern and clinical significance of DNM2 in bladder cancer by applying available clinicopathological and follow‐up data obtained using IHC. However, to ensure the credibility of our findings, further studies should be conducted to confirm the nuclear localization of DNM2, such as employing fractional western blot analysis.

Most remarkably, the univariate analysis data showed an association between nuclear DNM2 expression and worsened DSS in bladder cancer patients. In addition, BC patients who expressed nuclear DNM2 showed shorter 5‐year DSS in comparison to cases without nuclear expression. However, the multivariate test indicated no significant association. Since nuclear DNM2 expression was significantly associated with the recurrence of bladder tumor and poor DSS in the univariate test, these findings could be because of the short‐term follow‐up and small patient population.

The mechanism underlying the nuclear expression of DNM2 is unknown. According to Reactome Pathway Database, hub genes involving DNM2 are clustered and involved in various pathways, such as “signaling by receptor tyrosine kinases”, “signaling by NTRK1 (TRKA)”, “signal transduction”, “Clathrin‐mediated endocytosis”, “L1CAM interactions”, “signaling by NTRKs” as summarized in Figure 3B. In addition to the above‐mentioned pathways, “signaling events mediated by VEGFR1 and VEGFR2”, “PDGFB signaling pathway”, “signaling by NGF”, “recycling pathway of cell adhesion molecule L1”, and “membrane trafficking” were considered as significantly enriched pathways by BioPlanet resource as shown in Figure 3C. DNM2 has participated in important signaling pathways with a high confidence score, which dysregulation and activations of these pathways play pivotal functions in carcinogenesis and tumor development. The majority of these signaling pathways are involved in regulating a wide variety of biological procedures, including cell growth, differentiation, motility, and angiogenesis, and are frequently deregulated in different malignancies. Targeting these pathways has been a fruitful therapeutic strategy in cancer [52]. We hypothesized that, according to the STRING‐PPI network and literature review, DNM2 is needed for the endocytosis of various proteins involved in tumor motility and invasiveness and for endocytosis of various oncogenic receptors, including the epidermal growth factor receptor (EGFR) [53], ErbB2 [54], and platelet‐derived growth factor receptor (PDGFR) [55]. It has been revealed that nuclear localization of ErbB2 implicates interaction with nuclear pore protein Nup358, the transport receptor importin‐1, and lots of players in endocytic internalization such as DNM2, as confirmed by electron microscopy and confocal immunofluorescence in MCF7/HER18 and MDA‐MB‐453 [56]. These results propose that importin‐1 and ErbB‐2 form complexes with multiple accessory endocytic proteins, including EPS15 and DNM2, transported from cytoplasmic to the nuclear compartment of the tumor cells. DNM2 appears to be a key factor governing cellular reprogramming toward cancerous through endocytosis and transferring of various oncogenic molecules to the nucleus, as expression of defective DNM2 inhibited EGFR nuclear transport [56, 57]. These nuclear receptor tyrosine kinases (RTKs) have been revealed to function as transcription factors (TF) or transcriptional coactivators for oncogenic genes like FGF2 and c‐myc [58, 59, 60]. Therefore, the nuclear DNM2 expression in bladder cancer supports the aggressive nature and higher stages of the tumor. On the other hand, it has been reported that c‐Myc and ERBB2 cooperate to drive a stem‐like phenotype and a mesenchymal‐like invasive state [61], which probably confirmed our results regarding the association of nuclear DNM2 expression and higher grade. However, further studies should be done to elucidate the molecular mechanisms of the DNM2 nuclear localization and the following signaling pathways in bladder cancer.

The study is restricted by a small sample size, retrospective design, and the lack of functional analysis. Our results suggest that an elevated nuclear expression of DNM2 is associated with disease progression. Future investigations with a larger population and a prospective study design will provide a more comprehensive understanding of the expression of DNM2 and contribute to the advancement of our research. In addition, to enhance our knowledge, future research should prioritize investigating the effects of DNM2 downregulation through siRNA‐mediated knockdown or CRISPR/Cas9‐mediated knockout in bladder tumors. This analysis would shed light on the impact of DNM2 on pluripotency, metastasis, and migration. Another restriction was that we could not find pT3 and pT4 stages in our cohort, as most patients are nonmuscle invasive bladder cancer. Furthermore, it is crucial to conduct experimental studies to investigate the mechanisms in which DNM2 is involved, as identified through the results of our bioinformatics analysis.

## 5 Conclusion

In conclusion, our data mining findings using various bioinformatics tools demonstrated dysregulation of DNM2 mRNA expression in bladder tumor compared to normal tissue samples adjacent to tumor and its involvement in different signaling pathways. The findings of our IHC study exhibit a potential capacity of nuclear DNM2 in the prediction of poor clinicopathological outcomes in bladder cancer cases. The current study indicates that nuclear DNM2 protein expression, in comparison with membranous and cytoplasmic expression, plays a key role in the more aggressive carcinoma nature and development of BC. Further, bladder tumor patients with nuclear DNM2 overexpression had shorter DSS, suggesting that nuclear expression of DNM2 may represent an important biological marker. Consequently, evaluating the DNM2 expression in different subcellular localization is valuable for the prediction of cancer invasiveness and prognosis. However, further large‐scale investigations are required to identify the importance of DNM2 prognostic and molecular mechanisms in presenting novel opportunities for therapeutic targeting of BC.

## Author Contributions

**Mahdieh Razmi:** investigation, writing–original draft, methodology, visualization, data curation, formal analysis. **Leili Saeednejad Zanjani:** formal analysis, methodology. **Mandana Rahimi:** validation, methodology, data curation. **Roya Sajed:** writing–original draft, visualization. **Sadegh Safaei:** writing–original draft, visualization. **Zahra Madjd:** conceptualization, writing–review and editing, supervision. **Roya Ghods:** conceptualization, funding acquisition, writing–review and editing, supervision.

## Ethics Statement

This study was approved by the Human Research Ethics Committee of Iran University of Medical Sciences in Iran (No. 98‐04‐28‐17448). All methods were performed in accordance with the relevant guidelines and regulations. Informed consent was received from each patient participating in the research. After the collection of clinical information, patient identifiers were removed, and subsequently, patients could not be directly or indirectly identified.

## Conflicts of Interest

The authors declare no conflicts of interest.

## Supporting information


**Figure S1.** Second cluster subnetwork was identified from the PPI network with the help of Cytoscape using the MCODE plugin.

**Figure S2.** Subcellular localization assessment of DNM2.

**Table S1.** Investigation of dynamin 2 (DNM2) on the Gene Expression database of Normal and Tumor tissues 2 (GENT2) database for bladder cancer.

**Table S2.** The association between cytoplasmic dynamin 2 (DNM2) expression and clinicopathological characteristics in bladder carcinoma (Intensity of staining and H‐score).

**Table S3.** The association between membranous dynamin 2 (DNM2) expression and clinicopathological characteristics in bladder carcinoma (Intensity of staining and H‐score).

## Data Availability

The raw data are available from the corresponding author on reasonable request. All datasets were public datasets and were freely available. The datasets generated and/or analyzed during the current study are available in the [GEPIA2] repository (http://gepia2.cancer‐pku.cn/#degenes), the [GENT2] repository (http://gent2.appex.kr), the [UALCAN] repository (http://ualcan.path.uab.edu/), the [STRING] repository (https://string‐db.org/cgi/network?taskId=b2M35N7Ft9Cp&sessionId=b4CfoQV9svcu) and the [Enrichr] repository (amp.pharm.mssm.edu/Enrichr/).
