# Supplementary material for: Nuclear Expression of Dynamin 2 Is Associated With Tumor Aggressiveness in Bladder Cancer Patients: A Bioinformatics and Experimental Approach
Source: Cancer Rep (Hoboken). 2024 Nov 28;7(12):e2133. doi: 10.1002/cnr2.2133 (PMC11604598; doi:10.1002/cnr2.2133)
Supplement: Supplementary file 1 — Figure S1. Second cluster subnetwork was identified from the PPI network with the help of Cytoscape using the MCODE plugin. [file CNR2-7-e2133-s005.docx]

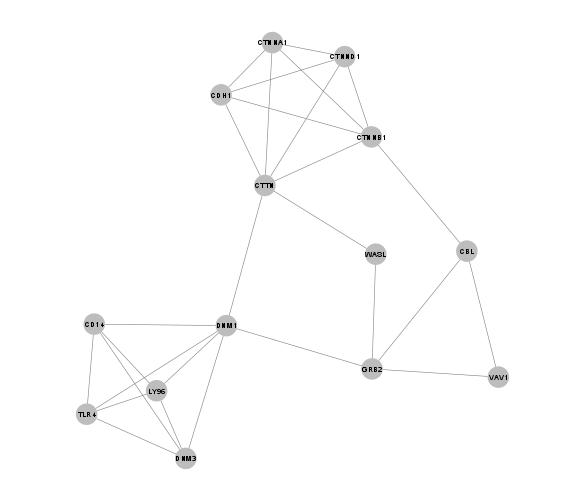


**Figure S1**. Second cluster subnetwork was identified from the PPI network with the help of Cytoscape using the MCODE plugin
