# Supplementary figures and images for: Nuclear Expression of Dynamin 2 Is Associated With Tumor Aggressiveness in Bladder Cancer Patients: A Bioinformatics and Experimental Approach
Source: Cancer Rep (Hoboken). 2024 Nov 28;7(12):e2133. doi: 10.1002/cnr2.2133 (PMC11604598; doi:10.1002/cnr2.2133)

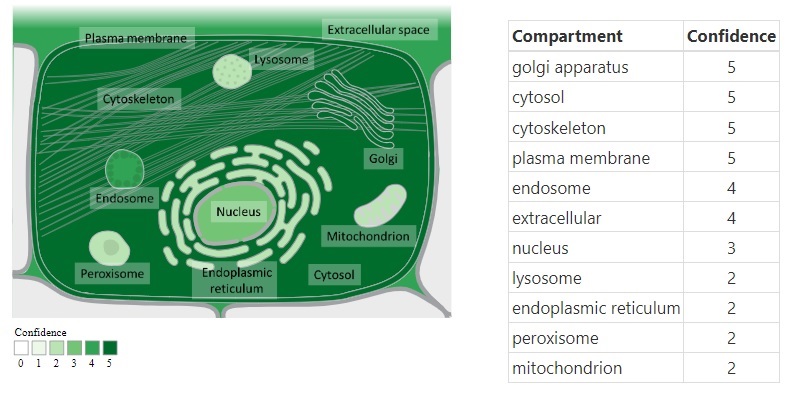


**Figure S2**. Subcellular localization assessment of DNM2.

Supplement: Supplementary file 2 — Figure S2. Subcellular localization assessment of DNM2. [file CNR2-7-e2133-s004.docx]
