# Supplementary material for: Nuclear Expression of Dynamin 2 Is Associated With Tumor Aggressiveness in Bladder Cancer Patients: A Bioinformatics and Experimental Approach
Source: Cancer Rep (Hoboken). 2024 Nov 28;7(12):e2133. doi: 10.1002/cnr2.2133 (PMC11604598; doi:10.1002/cnr2.2133)
Supplement: Supplementary file 3 — Table S1. Investigation of dynamin 2 (DNM2) on the Gene Expression database of Normal and Tumor tissues 2 (GENT2) database for bladder cancer. [file CNR2-7-e2133-s003.docx]

| Microarray platforms | P-value | Log_2_FC |
| --- | --- | --- |
| GPL570 platform (HG-U133_Plus_2) | < 0.001 | 0.607 |
| GPL96platform (HG-U133A)] | 0.297 | -0.219 |

**Table S1**. Investigation of dynamin 2 (DNM2) on the Gene Expression database of Normal and Tumor tissues 2 )GENT2( database for bladder cancer.
