# Supplementary material for: Nuclear Expression of Dynamin 2 Is Associated With Tumor Aggressiveness in Bladder Cancer Patients: A Bioinformatics and Experimental Approach
Source: Cancer Rep (Hoboken). 2024 Nov 28;7(12):e2133. doi: 10.1002/cnr2.2133 (PMC11604598; doi:10.1002/cnr2.2133)
Supplement: Supplementary file 4 — Table S2. The association between cytoplasmic dynamin 2 (DNM2) expression and clinicopathological characteristics in bladder carcinoma (Intensity of staining and H‐score). [file CNR2-7-e2133-s002.docx]

**Table S2**. The association between cytoplasmic dynamin 2 (DNM2) expression and clinicopathological characteristic in bladder carcinoma (Intensity of staining and H-score)

| **Patients and tumor characteristics** | **Total no.**  **cases** | **Intensity of staining N (%)** | | | | ***P-* *value*** | **H-score (cut off = 285) N (%)** | | ***P-* *value*** |
| --- | --- | --- | --- | --- | --- | --- | --- | --- | --- |
|  |  | **0 (Negative)** | **1+**  **(Weak)** | **2+**  **(Moderate)** | **3+ (Strong)** |  | **Low (≤285)** | **High (>285)** |  |
| Bladder carcinoma tissues | 209 (100) | 2 (1.0) | 25 (12.0) | 43 (20.5) | 139 (66.5) |  | 119 (56.9) | 90 (43.1) |  |
| **Median age (years)** | | | | | | | | | |
| 66 ≤ | 106 (50.7) | 2 (100) | 13 (52.0) | 22 (51.2) | 69 (49.6) | 0.567 | 63 (52.9) | 43 (47.8) | 0.460 |
| 66 > | 103 (49.3) | 0 (0.0) | 12 (48.0) | 21 (48.0) | 70 (50.4) |  | 56 (47.1) | 47 (52.2) |  |
| **Gender** | | | | | | | | | |
| Male | 156 (74.6) | 1 (50.0) | 20 (80.0) | 32 (74.4) | 103 (74.1) | 0.791 | 90 (75.6) | 66 (73.3) | 0.705 |
| Female | 53 (25.4) | 1 (50.0) | 5 (20.0) | 11 (25.6) | 36 (25.9) |  | 29 (24.4) | 24 (26.7) |  |
| **Tumor size (cm)** | | | | | | | | | |
| 2.4 ≤ | 128 (61.2) | 1 (50.0) | 18 (72.0) | 28 (65.1) | 81 (58.3) | 0.549 | 77 (64.7) | 51 (56.7) | 0238 |
| 2.4 > | 81 (38.8) | 1 (50.0) | 7 (28.0) | 15 (34.9) | 58 (41.7) |  | 42 (35.3) | 39 (43.3) |  |
| **Histological grade** | | | | | | | | | |
| Low | 126 (60.3) | 0 (0.0) | 13 (52.0) | 26 (60.5) | 87 (62.6) | 0.255 | 70 (58.8) | 56 (62.2) | 0.619 |
| High | 83 (39.7) | 2 (100) | 12 (48.0) | 17 (39.5) | 52 (37.4) |  | 49 (41.2) | 34 (37.8) |  |
| **pT stage** | | | | | | | | | |
| pTa | 120 (57.4) | 1 (50) | 11 (44) | 23 (53.5) | 85 (61.2) | 0.239 | 65 (54.6) | 55 (61.1) | 0.545 |
| pT1 | 71 (34.0) | 0 (0.0) | 12 (48.0) | 16 (37.2) | 43 (30.9) |  | 42 (35.3) | 29 (32.2) |  |
| pT2 | 18 (8.6) | 1 (50) | 2 (8) | 4 (9.3) | 11 (7.9) |  | 12 (10.1) | 6 (6.7) |  |
| pT3 | 0 (0.0) | 0 (0.0) | 0 (0.0) | 0 (0.0) | 0 (0.0) |  | 0 | 0 |  |
| pT4 | 0 (0.0) | 0 (0.0) | 0 (0.0) | 0 (0.0) | 0 (0.0) |  | 0 | 0 |  |
| **Muscularis invasion** | | | | | | | | | |
| Involved | 18 (8.6) | 1 (50) | 2 (8) | 4 (9.3) | 11 (7.9) | 0.214 | 12 (10.1) | 6 (6.7) | 0.383 |
| None | 191 (91.4) | 1 (50) | 23 (92) | 39 (90.7) | 128 (92.1) |  | 107 (89.9) | 84 (93.3) |  |
| **Tumor recurrence** | | | | | | | | | |
| Present | 41 (19.6) | 1 (50) | 6 (24) | 9 (20.9) | 25 (18) | 0.624 | 23 (19.3) | 18 (20.0) | 0.904 |
| Absent | 168 (80.4) | 1 (50) | 19 (76) | 34 (79.1) | 114 (82) |  | 96 (80.7) | 72 (80.0) |  |
| **Distant metastasis** | | | | | | | | | |
| Present | 25 (12) | 1 (50) | 5 (20) | 3 (7) | 16 (11.5) | 0.150 | 14 (11.8) | 11 (12.2) | 0.920 |
| Absent | 184 (88) | 1 (50) | 20 (80) | 40 (93) | 123 (88.5) |  | 105 (88.2) | 79 (87.8) |  |
| H-score indicates histological score.  *P* *value*; Pearson's χ2 test.  Values in bold are statistically signiﬁcant. | | | | | | | | | |
