# Supplementary material for: Nuclear Expression of Dynamin 2 Is Associated With Tumor Aggressiveness in Bladder Cancer Patients: A Bioinformatics and Experimental Approach
Source: Cancer Rep (Hoboken). 2024 Nov 28;7(12):e2133. doi: 10.1002/cnr2.2133 (PMC11604598; doi:10.1002/cnr2.2133)
Supplement: Supplementary file 5 — Table S3. The association between membranous dynamin 2 (DNM2) expression and clinicopathological characteristics in bladder carcinoma (Intensity of staining and H‐score). [file CNR2-7-e2133-s001.docx]

**Table S3**. The association between membranous dynamin 2 (DNM2) expression and clinicopathological characteristic in bladder carcinoma (Intensity of staining and H-score)

| **Patients and tumor characteristics** | **Total no.**  **cases** | **Intensity of staining N (%)** | | | | ***P-* *value*** | **H-score (cut off = 150) N (%)** | | ***P-* *value*** |
| --- | --- | --- | --- | --- | --- | --- | --- | --- | --- |
|  |  | **0 (Negative)** | **1+**  **(Weak)** | **2+**  **(Moderate)** | **3+ (Strong)** |  | **Low (≤150)** | **High (>150)** |  |
| **Bladder carcinoma tissues** | 209 | 27 (12.9) | 17 (8.1) | 47 (22.5) | 118 (56.5) |  | 108 (51.7) | 101 (48.3) |  |
| **Median age (years)** | | | | | | | | | |
| 66 ≤ | 106 (50.7) | 14 (51.9) | 9 (52.9) | 25 (53.2) | 58 (49.2) | 0.964 | 59 (54.6) | 47 (46.5) | 0.242 |
| 66 > | 103 (49.3) | 13 (48.1) | 8 (47.1) | 22 (46.8) | 60 (50.8) |  | 49 (45.4) | 54 (53.5) |  |
| **Gender** | | | | | | | | | |
| Male | 156 (74.6) | 20 (74.1) | 15 (88.2) | 33 (70.2) | 88 (74.6) | 0.542 | 82 (75.9) | 74 (73.3) | 0.659 |
| Female | 53 (25.4) | 7 (25.9) | 2 (11.8) | 14 (29.8) | 30 (25.4) |  | 26 (24.1) | 27 (26.7) |  |
| **Tumor size (cm)** | | | | | | | | | |
| 2.4 ≤ | 128 (61.2) | 18 (66.7) | 10 (58.8) | 31 (66) | 69 (58.5) | 0.754 | 70 (64.8) | 58 (57.4) | 0.273 |
| 2.4 > | 81 (38.8) | 9 (33.3) | 7 (41.2) | 16 (34) | 49 (41.5) |  | 38 (35.2) | 43 (42.6) |  |
| **Histological grade** | | | | | | | | | |
| Low | 126 (60.3) | 12 (44.4) | 8 (47.1) | 34 (72.3) | 72 (61) | 0.073 | 63 (58.3) | 63 (62.4) | 0.551 |
| High | 83 (39.7) | 15 (55.6) | 9 (52.9) | 13 (27.7) | 46 (39) |  | 45 (41.7) | 38 (37.6) |  |
| **pT stage** | | | | | | | | | |
| pTa | 120 (57.4) | 12 (44.4) | 6 (35.3) | 30 (63.8) | 72 (61) | 0.068 | 58 (53.7) | 62 (61.4) | 0.477 |
| pT1 | 71 (34.0) | 14 (51.9) | 7 (41.2) | 14 (29.8) | 36 (30.5) |  | 39 (36.1) | 32 (31.7) |  |
| pT2 | 18 (8.6) | 1 (3.7) | 4 (23.5) | 3 (6.4) | 10 (8.5) |  | 11 (10.2) | 7 (6.9) |  |
| pT3 | 0 (0.0) | 0 (0.0) | 0 (0.0) | 0 (0.0) | 0 (0.0) |  | 0 (0.0) | 0 (0.0) |  |
| pT4 | 0 (0.0) | 0 (0.0) | 0 (0.0) | 0 (0.0) | 0 (0.0) |  | 0 (0.0) | 0 (0.0) |  |
| **Muscularis invasion** | | | | | | | | | |
| Involved | 18 (8.6) | 1 (3.7) | 4 (23.5) | 3 (6.4) | 10 (8.5) | 0.115 | 11 (10.2) | 7 (6.9) | 0.402 |
| None | 191 (91.4) | 26 (96.3) | 13 (76.5) | 44 (93.6) | 108 (91.5) |  | 97 (89.8) | 94 (93.1) |  |
| **Tumor recurrence** | | | | | | | | | |
| Present | 41 (19.6) | 2 (7.4) | 8 (47.1) | 11 (23.4) | 20 (16.9) | 0.092 | 24 (22.2) | 17 (16.8) | 0.327 |
| Absent | 168 (80.4) | 25 (92.6) | 9 (52.9) | 36 (76.6) | 98 (83.1) |  | 84 (77.8) | 84 (83.2) |  |
| **Distant metastasis** | | | | | | | | | |
| Present | 25 (12) | 1 (3.7) | 5 (29.4) | 5 (10.6) | 14 (11.9) | 0.081 | 15 (13.9) | 10 (9.9) | 0.375 |
| Absent | 184 (88) | 26 (96.3) | 12 (70.6) | 42 (89.4) | 104 (88.1) |  | 93 (86.1) | 91 (90.1) |  |
| H-score indicates histological score.  *P* *value*; Pearson’s χ2 test.  Values in bold are statistically signiﬁcant. | | | | | | | | | |
